# The Role of Estradiol in Traumatic Brain Injury: Mechanism and Treatment Potential

**DOI:** 10.3390/ijms22010011

**Published:** 2020-12-22

**Authors:** Erzsébet Kövesdi, Edina Szabó-Meleg, István M. Abrahám

**Affiliations:** 1 Molecular Neuroendocrinology Research Group, Institute of Physiology, Medical School, Center for Neuroscience, Szentágothai Research Center, University of Pécs, H-7624 Pecs, Hungary; kovesdi.erzsebet@pte.hu; 2 Department of Biophysics, Medical School, University of Pécs, H-7624 Pecs, Hungary; edina.meleg@aok.pte.hu

**Keywords:** 17β-estradiol, traumatic brain injury, rodents, human, neuroprotection, treatment

## Abstract

Patients surviving traumatic brain injury (TBI) face numerous neurological and neuropsychological problems significantly affecting their quality of life. Extensive studies over the past decades have investigated pharmacological treatment options in different animal models, targeting various pathological consequences of TBI. Sex and gender are known to influence the outcome of TBI in animal models and in patients, respectively. Apart from its well-known effects on reproduction, 17β-estradiol (E2) has a neuroprotective role in brain injury. Hence, in this review, we focus on the effect of E2 in TBI in humans and animals. First, we discuss the clinical classification and pathomechanism of TBI, the research in animal models, and the neuroprotective role of E2. Based on the results of animal studies and clinical trials, we discuss possible E2 targets from early to late events in the pathomechanism of TBI, including neuroinflammation and possible disturbances of the endocrine system. Finally, the potential relevance of selective estrogenic compounds in the treatment of TBI will be discussed.

## 1. Introduction

Traumatic brain injury (TBI) is defined as an alteration in brain function or any other evidence of brain pathology caused by an external force [1]. Importantly, TBI has become one of the leading causes of mortality and morbidity. However, the exact number of TBI cases per year is not known, due to many undiagnosed cases who do not receive medical care. The estimated incidence of hospitalized TBI patients is enormous, with an annual incidence of approximately 235/100,000 in the European Union [2], further associated with a cost of 33 billion euro [3].

Appropriate emergency medicine and intensive care reduce the number of deaths [4]; however, patients who survive TBI face numerous neurological and neuropsychological problems [5], such as cognitive, motor, and mental disturbances, which significantly affect their quality of life [6]. In most cases, the therapeutic TBI intervention involves surgical removal of hematomas and skull fractures, along with medical therapies to maintain proper cerebral perfusion and oxygenation of the brain tissue. In patients not needing surgery, the control of intracranial pressure (ICP) and cerebral perfusion pressure (CPP) are essential during the recovery phase in intensive care units. Since the primary injury itself cannot be treated, only secondary injury can be prevented or reduced; thus, experimental neuroprotective interventions focus mainly on the secondary injury. Several pharmacological treatment options have been tested in animal models, targeting the varied pathological consequences of TBI. More than 130 monotherapies seemed like promising neuroprotective agents in animal models [7] but failed in clinical trials. Based on the United States Government database (www.clinicaltrials.gov), 1308 clinical trials are still active or completed worldwide. From these trials, 867 addressed different therapeutic options but only seven dealt with clinical testing of neuroprotective agents. Therefore, to date, there is still no approved therapy for TBI [8,9].

The outcome of TBI is sexually dimorphic in animal models and sex-dependent in patients with severe TBI. Several clinical studies have demonstrated that males have higher mortality rates and higher incidence of complications than females [10,11,12], suggesting that gonadal steroids such as 17β-estradiol (E2) play a critical role in the outcome of TBI. Animal studies have demonstrated the remarkable neuroprotective potential of E2 [13,14,15,16]. However, clinical trials have shown conflicting results regarding the effectiveness of female sexual hormones in TBI treatment [17,18].

In recent years, several research groups demonstrated the effect of E2 as a possible therapeutic agent of TBI [19,20,21,22]. Accordingly, the aim of this review is to summarize the role and mechanism of action of E2 in TBI. First, we review the clinical aspects, pathomechanism of TBI, animal models, and E2-induced neuroprotective effects. Second, we discuss the possible mechanisms of E2 action targeting neuropathological events in TBI, such as disruption of the blood–brain barrier (BBB), mitochondrial dysfunction, free radicals, neuroinflammatory cascade, calpain activity, apoptotic cell death, and disturbance of the endocrine system, including hypothalamic pituitary-gonadal axis damage. Finally, based on the findings of preclinical rodent and human clinical studies, the relevance and potential clinical application of E2 and estrogenic compounds in the treatment of TBI will be summarized.

## 2. TBI Classification

Head injuries are classified into two major categories: primary and secondary injuries [23]. Based on the physical intervention, primary injuries can be further classified as penetrating (open-head) or nonpenetrating (closed-head). According to the neuroanatomical location, primary injuries can be focal or diffuse.

Focal injuries can be found in most forms of severe and moderate TBI. Contusion, hemorrhage, intracranial bleedings, including subarachnoid hemorrhage, and subdural hematoma are the typical forms of focal injury. While coup injuries occur at the side, the contrecoup are on the opposite side of the impact. Subarachnoid hemorrhage is the most common form of vascular injury in TBI [24]. Hemorrhage within the contusion area leads to the formation of local edema and ischemia and results in tissue destruction and neuronal necrosis [25]. Subdural hematomas are the result of ruptured veins due to rapid acceleration-deceleration forces during TBI [26,27]. Focal lesions are simple to identify by routine imaging techniques while promising blood biomarkers such as the glial fibrillary acidic protein (GFAP) and its breakdown products allow recognizing hemorrhagic lesions in very early stages, even in patients with relatively mild injury [28,29,30]. While these lesions mainly involve the frontal and temporal lobes, the subcortical white matter can also be affected in more severe forms of TBI. Hemorrhage within the contusion area leads to local edema formation and ischemia and results in tissue destruction and neuronal necrosis [25].

Diffuse injuries occur in a scattered form within the affected neuronal and vascular structures. Forms of diffuse injury include microvascular and axonal injuries [26]. Diffuse axonal injuries occur both in gray and white matter both in humans and animals in all severity ranges and forms of TBI, involving diffuse neuronal damage, microvascular change, axonal perturbation, and eventually, axon disconnection. Then, the distal, detached axonal projections undergo target deafferentation and synaptic loss with Wallerian degeneration [25].

## 3. Pathomechanism of TBI

**Figure 1:**
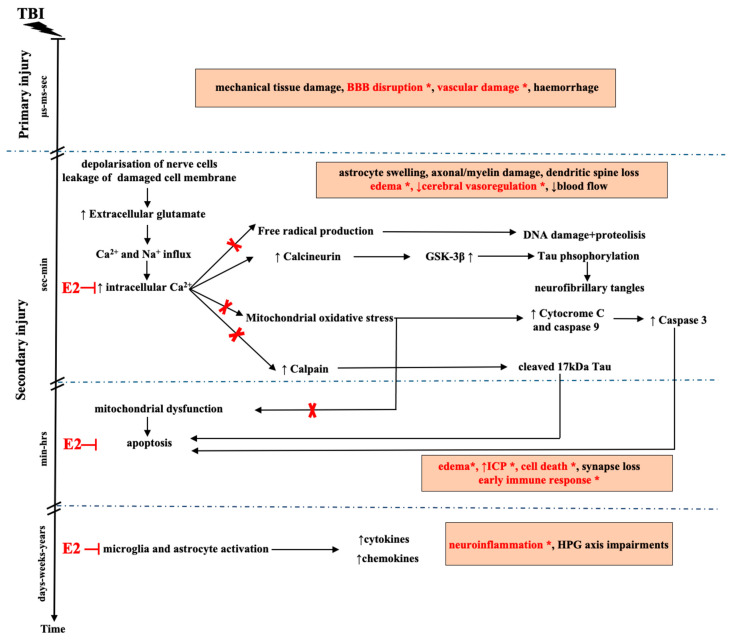
Timeline of events after the TBI and proposed neuroprotective effects of 17β-estradiol (E2). Red “X” or horizontal “T” letter and “*” with red letters depict blocked or reduced processes in the TBI pathomechanism after E2 administration. ↑: increased, ↓: decreased. Abbreviations: BBB: blood–brain barrier, GSK3β: glycogen synthase kinase 3 beta, HPG: hypothalamic-pituitary-gonadal, ICP: intracranial pressure, and TBI: traumatic brain injury.

Primary injuries are the result of external mechanical forces that produce irreversible tissue damage at the moment of the injury, lasting from microseconds to seconds (Figure 1). Importantly, in primary injuries, several neuropathological processes emerge: mechanical tissue destruction, cell and axonal stretching, blood–brain barrier (BBB) disruption, synaptic loss, the failure of ATP-dependent glutamate transporters, and damage to blood vessels [31,32] (Figure 1). Excessive glutamate release after TBI is one of the key factors in TBI’s pathomechanism. Elevated glutamate levels originate from damaged neurons and glia, from presynaptic vesicles of depolarized neurons, or from the damaged BBB [33] (Figure 1). The increased glutamate level correlates with injury severity and unfavorable outcomes [34,35]. Based on clinical studies, the glutamate level is highest immediately after injury, which can last one to two days post-TBI. Elevated glutamate levels lead to N-methyl-D-aspartic acid (NMDA) and α-amino-3-hydroxy5-methyl-4-isoxazole-propionic acid (AMPA) receptor activation [36] and the consequent accumulation of extracellular K^+^ and Ca^2+^ and Na^+^ influx [37,38,39]. Ca^2+^ ions also enter cells via voltage-gated calcium channels (VGCCs) [40,41] and the ionotropic P2X purinoceptor 7 (P2X7) [42]. Disturbed Ca^2+^ homeostasis triggers several parallel pathological events, such as mitochondrial dysfunction, oxidative stress, cerebral edema, and inflammatory cascades of the secondary injury (Figure 1). The secondary injury occurs from seconds to days from the time of the primary injury and influences the outcome and recovery [43,44]. Increased intracellular Ca^2+^ levels promote free-radical production and activate calcineurin, endonucleases, cytochrome C, and calpain/caspase-mediated spectrin proteolysis, resulting in mitochondrial oxidative stress and apoptosis [45,46]. In addition, excess Ca^2+^ levels induce calpain and calcineurin activation [47,48], which play a role in the formation of neurofibrillary tangles via cleavage-induced GSK-3β activation. This process leads to hyperphosphorylated tau [49], microtubule disruption, and tau accumulation [50,51].

Neuronal mitochondria with high Ca^2+^ levels are targets for free radicals (Figure 1). The production of free radicals and the imbalance between oxidant and antioxidant factors after TBI promote the development of cerebral edema and increased ICP [52]. With the disrupted BBB, the increased ICP leads to immune cell entry, inducing oxidative damage and cell death through the elevated levels of various cytokines and chemokines [53]. Early neuroinflammatory responses begin a few hours after TBI and last from weeks to months due to microglia activation [54] and the increased infiltration of neutrophils, macrophages, and gliosis around the affected brain area [55] (Figure 1).

## 4. Animal Models of TBI

Animal studies enable to explore the effect of TBI on various aspects like sex differences, age, onset of injury, type, and severity. However, modeling TBI is a major challenge for neuroscientists. To date, there is no comprehensive experimental model of TBI able to represent all aspects of the injury. Experimental focal injury models include the controlled cortical impact (CCI), midline/lateral fluid percussion injury (FPI), controlled concussion, and weight-drop models by Feeney [56] and Shohami [57]. The experimental diffuse injury models are Marmarou’s impact acceleration model, diffuse brain injury, and inertial acceleration models [58]. The most frequently used animal models are the CCI, FPI, and Marmarou’s model, which can mimic the pathophysiology and cognitive deficits of TBI [59]. CCI is used mostly in mouse experiments, while the other two are used in rats. 

In the CCI model, the brain is impacted through a surgically exposed dura by a rapidly accelerated rod, which directly impacts the brain tissue [60]. The injury causes acute hematoma, BBB damage, cortical contusion, deep brain injury, and neurodegeneration around the impacted area [61]. The CCI model was further developed for closed skull injury, enabling the analysis of mild and repetitive TBI [61,62].

The FPI mimics human TBI without skull fracture [63], but it induces contusion, brain tissue stretching, subdural hematoma, and hemorrhage [64]. In the FPI, the intact dura is exposed through a needle (luer lock hub), and the injury is induced through this craniotomy by a pendulum striking the piston at the end of a tube filled with liquid. Based on the height of the pendulum, the injury severity can vary between mild and severe injuries. FPI primarily results in contusion, subdural hematoma, tissue shearing/stretching, and necrotic cell death. Secondary events include the activation of inflammatory glial cells and neuronal cell death [64]. It is interesting that the same device can be used for diffuse and mixed (focal and diffuse) injury: upper mild, moderate, and severe pressure impulses cause focal and diffuse injury, while lower mild pressure produces diffuse injury [65]. Based on the location of the craniotomy, there are two main FPI types: midline (MFP) and lateral (LFP). In MFP, the craniotomy is at the center of the sagittal suture, while, in the LFP, the skull window is performed 3.5 mm lateral to the midline [66]. LFP is an appropriate model for mimicking the histopathology and behavioral outcomes of TBI. In LFP, focal cortical contusion and the diffuse subcortical neuronal injury of the injured brain side are minimally transferred to the noninjured contralateral side [67], so the contralateral side can serve as a control to evaluate the damage caused by the injury.

Marmarou’s impact acceleration model mimics the human diffuse TBI resulting from falls or motor vehicle accidents [68]. A metal disc is used in this model, which is placed on the skull and protects the animal from bone fractures. Brain tissue deformation and injury severity can vary between mild and severe injuries based on the height of the falling impactor. The model can result in cell loss, diffuse axonal injury, astrogliosis, and hemorrhage [69].

Other weight-drop models include Feeney’s, Shapira’s, and Shohami’s TBI models. In Feeney’s model, the injury is caused by a falling weight through a craniotomy onto the intact dura. This injury causes cortical contusion [56,70], hemorrhage [71], and BBB damage [72,73]. In contrast to Marmarou’s model, Shapira and Shohami’s models use head fixation to avoid acceleration diffuse injury, such as that observed in Marmarou’s model [57,74].

## 5. E2 and Neuroprotection

In the hypothalamo-pituitary-gonadal (HPG) axis, the central regulator of fertility, the hypothalamic gonadotropin-releasing hormone (GnRH) neurons control the synthesis and pulsatile secretion of the luteinizing hormone (LH) and follicle-stimulating hormone (FSH) [75] in the anterior lobe of the pituitary. The kisspeptin, neurokinin B, and dynorphin (KNDy) neurons of the arcuate nucleus co-expressing kisspeptin/neurokinin B/dynorphin are the main regulators of episodic GnRH release. In these neurons, neurokinin B initiates the pulse onset, kisspeptin drives GnRH secretion, and dynorphin terminates the pulse [76]. The released LH and FSH increase ovarian E2 synthesis and folliculogenesis during the estrus cycle [77]. In males, E2 is produced from testosterone via aromatase in adipocytes, bones, and the brain [78]. The E2 level in men is low, very similar to that in postmenopausal women [79].

In females, E2 has an important regulatory effect on the reproductive processes as a positive and negative feedback regulator [80,81]. E2 exerts a positive feedback action on GnRH neurons via kisspeptin neurons, inducing GnRH secretion, LH surge, and consequently ovulation. In male rodents, testosterone continuously inhibits the pulsatile function of GnRH neurons. In rodents, the pulsatile activity of GnRH neurons is controlled by a E2 negative feedback, with E2 inhibiting GnRH neurons for most of the estrus cycle [82].

In the brain, E2 originates from a variety of sources, including the gonads, local conversion of circulating androgen precursors via aromatase, and direct synthesis from cholesterol in neurons and the glia [83]. During brain injuries such as TBI, aromatase expression is induced in astrocytes [84,85,86], representing a mechanism of neuroplasticity activated for repair [87]. Brain steroidogenesis is independently regulated from peripheral steroidogenesis [88], because the enzymes responsible for E2 biosynthesis are present in the brain and expressed in both neurons and astrocytes [89,90].

E2 exerts its effects through two classical estrogen receptors (ERs), estrogen receptor alpha (ERα) and beta (ERβ) [91,92,93]. ERs are ligand-activated transcription factors in tissues that play a role in reproductive functions, and several tissues and organs, such as the brain, cardiovascular tissue, bone, immune cells, and liver express ER [94]. In the plasma membrane, ERs may be located in lipid rafts [95], allowing the direct interaction of ERs with different signaling pathways [96]. In the brain, ERs are expressed by neurons, microglia, astrocytes, oligodendrocytes, endothelial cells, and smooth muscle cells in blood vessels [97] and brain regions that play important roles in learning and memory processes, such as the amygdala, cerebral cortex, hippocampus, and basal forebrain [98]. The cellular effects of E2 occur through “classical” genomic and nonclassical signaling pathways. E2 effects via the “classical” pathway are slow and can last from hours to days. E2 binds to ERα or ERβ and triggers phosphorylation and dimerization of the ER-E2 complex, as well as the removal of regulatory receptor-associated proteins [99] and attachment of coactivators [100,101]. The dimerized complex translocates into the nucleus and binds to the estrogen response element (ERE), promoting the recruitment of specific coregulators that modulate the expression of target genes [102].

E2 exerts its rapid nonclassical effect on membrane receptors and intracellular signaling molecules. One of the targets of nonclassical E2 action is the classical ER associated with the membrane via scaffolding proteins such as caveolin-1 [96]. Importantly, E2 can bind to membrane receptors such as the G protein-coupled estrogen receptor 1 (GPER1) [103]. Binding to cytoplasmic ER or GPER1, E2 induces ERE-independent gene transcription by activating different intracellular second messenger pathways, including mitogen-activated protein kinase (MAPK) [104], phosphatidylinositol 3-kinase (PI3K) [105,106], and protein kinases A and C [107]. The nonclassical effect of E2 also involves the rapid phosphorylation of transcription factors such as the cAMP-responsive element-binding protein (CREB) [108], which initiates indirect gene transcription [109] and the regulation of several neurotrophic and neuroprotective factors, including hypoxia-inducible factor 1α, brain-derived neurotrophic factor, vascular endothelial growth factor, transforming growth factor β, and glial derived neurotrophic factor [110]. In vitro studies in neuronal cultures demonstrated the neuroprotective effects of E2 against toxicity from serum deprivation [111,112], β-amyloid treatment [113,114], and oxidative stress [115,116]. In vivo brain injury models have also confirmed the beneficial effects of E2 in different ischemia and neurodegenerative animal models after middle cerebral artery occlusion [117,118,119] and photothrombotic focal ischemia [120], as well as in Parkinson’s [121,122], Alzheimer’s disease [123,124], and multiple sclerosis [125] animal models. In general, the nonclassical pathway is responsible for the neuroprotective effect of E2 [123,124].

Animal experiments demonstrated the neuroprotective potential of E2 in TBI. Ovariectomized (OVX) rats have a larger contusion volume immediately postinjury compared to intact rats [126]. A negative correlation between the circulating E2 level and the size of brain damage is also known to exist: in metestrus, the E2 level is low, and the brain lesion size larger than in proestrus with high E2 levels [127]. In the CCI model, animals had increased neurological severity scores, brain infarction size, and lasting brain edema [128]. Regarding cognitive functions, impaired acquisition and the retrieval of spatial memory in the Morris water maze (MWM) and increased anxiety levels in the elevated plus maze (EPM) were observed [128]. However, a daily intraperitoneal E2 treatment for seven days before or after TBI significantly reduced the neurological severity scores, brain infarction volume, edema, and apoptosis in the hippocampus [128]. The E2 treatment also improved the acquisition and retrieval of spatial memory in MWM and reduced anxiety levels in the EPM test [128].

## 6. Potential Targets of E2 Action in TBI

Several experiments demonstrated the protective mechanism of E2 in TBI. In vivo animal experiments on female rats showed that TBI significantly decrease the ERβ mRNA level, while the ERα mRNA level does not change [129]. E2 administration restored the ERβ mRNA levels to normal and increased the ERα mRNA (18%) and protein (35%) levels in the brain [129], suggesting that ERs may play a role in the E2-induced protective mechanism in TBI. The target and mechanisms of action of E2 involve cellular structures such as the BBB and mitochondria and molecular processes including intracellular Ca^2+^ homeostasis, calpain/caspase activity, free radical production, and neuroinflammatory processes (Figure 1).

### 6.1. E2 Effect on BBB and Mitochondria

E2 treatment enhances the cerebral blood flow and reduces the brain edema size and the ICP [130] after TBI, suggesting that E2 is capable of dampening the vascular damage and BBB disruption in TBI (Figure 1). Indeed, E2 can exert its neuroprotective effect via vasodilation and the decrease of vascular inflammation in the BBB. E2 increases the expression of endothelial nitric oxide synthase (eNOS) via the classical and nonclassical estrogen pathways in vascular endothelial cells, leading to brain vasodilation [131,132]. In the rat model of Marmarou, E2, acting through ERα and ERβ, decreased the edema and BBB disruption [133].

E2 also decreases inflammatory processes in endothelial cells by decreasing the expression of proinflammatory molecules such as e-selectin, intercellular adhesion molecule 1, and vascular cell adhesion molecule 1 [134] (Figure 1). Furthermore, E2 regulates the expression of tight junction proteins, playing an important role in the maintenance of the structure and integrity of the vascular endothelial membrane in capillaries [135,136]. These effects are mediated through the direct action of E2 on brain capillary endothelial cells [137] and indirectly through astrocytes and microglia.

Mitochondria-expressing ERs provide a platform for the neuroprotective effects of estrogens [138]. In vitro studies have demonstrated that E2 reduces mitochondrial dysfunction via the improvement of ATP production and moderation of intracellular Ca^2+^ levels during cellular stress to promote neuronal survival [139]. E2 maintains a normal level of ATP through increased oxidative phosphorylation and reduced ATPase activity and, thereby, increases the mitochondrial respiration efficiency and maintains normal mitochondrial functions. Furthermore, E2 increases the level of antiapoptotic proteins such as Bcl-2 and Bcl-xL to prevent the formation of a permeability transition pore (for review, see Nilsen et al. [140]).

### 6.2. Inhibition of TBI-Induced Intracellular Ca^2+^ Increase after E2 Application

Activity-induced presynaptic E2 production and release suggests that E2 may control the intracellular Ca^2+^ levels in neurons and astrocytes by regulating the neuronal excitability and preventing excitotoxicity and apoptosis [141,142] (Figure 1). Under pathological conditions, E2 is able to block the excessive increase of Ca^2+^ influx through L-type Ca^2+^ voltage-gated channels to protect motoneurons [143].

### 6.3. Effect of E2 on the Calpain/Caspase Activity in TBI

The modulation of apoptotic and necrotic cell death is a critical E2 cytoprotective mechanism, which may reduce the pathophysiological consequences of TBI [144,145]. There are two main mechanisms of neuronal cell death in brain injuries: apoptosis and necrosis [146]. Most apoptotic brain injuries can be either caspase-dependent or -independent. Mitochondria are essential for the activation of cell death signaling. Thus, the prevention of oxidative stress and the protection of normal mitochondrial functions result in decreased caspase activation and the inhibition of apoptosis [21]. In vitro studies demonstrated that E2 inhibits apoptosis in neurons exposed to glucose and oxygen deprivation [147], H_2_O_2_ [148,149], glutamate [143], β-amyloid [150], or neuroinflammatory molecules [151]. In a cortical contusion model (modified Feeney’s weight-drop model) [56], E2 protected the cells in the peri-contusional zone against apoptosis through ERα upregulation and blocking caspase-3 activation via the classical genomic pathway [152] (Figure 1).

### 6.4. Effect of E2 on Free Radical Production and Oxidative Stress after TBI

The brain tissue is very sensitive to damage induced by free radicals [153,154]. Several mechanisms may contribute to the antioxidant activity of E2: transcriptionally activates antioxidant enzymes and proteins and inhibits reactive oxygen species (ROS) production through the activation of mitochondrial antioxidative enzymes [155,156]. For instance, E2 prevents the ROS production induced by the mitochondrial toxin 3-nitropropionic acid [155]. E2 also inhibits the production of superoxide [157] and activates superoxide dismutase in vascular tissue via the activation of extracellular signal-regulated kinase 1/2 (ERK1/2) [157] and regulates catalase and glutathione peroxidase [158,159]. Further, E2 attenuates the H_2_O_2_-induced dose- and time-dependent decreases in cellular ATP production [155,160] (Figure 1).

### 6.5. E2 and Inflammation in TBI

The anti-inflammatory role of E2 was demonstrated in in vivo experimental models of Alzheimer’s disease [161], spinal cord injury [162], ischemic injury [163,164], and TBI [86,165]. In TBI, E2 treatment inhibits the production of inflammatory molecules such as tumor necrosis factor alpha, interleukin 1 beta, interleukin 6, transforming growth factor beta 1 [166], and prostaglandin E2 [21,167]. Furthermore, E2 suppresses the cyclooxygenase-2 pathway in cerebral blood vessels, preventing the migration of microglia into the affected brain area [168] and inhibiting proinflammatory gene expression through the regulation of nuclear factor kappa B [169,170] (Figure 1).

The E2-induced anti-inflammation gradually declines with age. Animal experiments demonstrated that the physiological concentration of E2 has anti-inflammatory activity in the brain in young OVX mice but not in older female rodents [171,172]. Suzuki and coworkers found that this anti-inflammatory effect of E2 disappears during prolonged hypoestrogenicity in middle-aged [173] or in “reproductively senescent” [174,175] mice.

## 7. TBI and the Impairment of the HPG Axis

Although the m part of our review focuses on the discussion of E2 action in TBI, clinical studies have demonstrated that TBI disturbs the HPG axis functions, including hormones such as E2. 

Animal experiments demonstrated HPG axis abnormalities after TBI. LFP-induced injuries result in anterior pituitary dysfunction, impaired estrous cycle, and reduced E2 and LH levels one week after TBI [176]. The decreased hormonal levels could be explained by the altered sensitivity to GnRH or dysregulated GnRH release. Injured rats showed impaired spatial working memory and sensorimotor functions. Reduced synaptic density in the hippocampus was also found one week after TBI, which can be explained by the possible consequences of HPG axis impairment.

Impairment and disruption of the HPG axis are very common in patients with severe TBI [177,178,179]. For instance, injuries affecting the anterior pituitary or the hypothalamus lead to GnRH dysregulation, altered LH and FSH release, and consequent impairment of E2 production [180]. Neuropathological processes, together with HPG damage, are linked to poor outcomes as a long-term consequence of the injury [181,182,183]. Although E2 has neuroprotective actions in males and females [127,184,185,186,187,188], the supra-physiological level of sex hormones at the time of severe TBI are usually markers of mortality or unfavorable outcomes in both genders, especially in people around and above 50 years of age [179,189]. Increased cortisol (CORT) and E2 levels are observed in TBI but, also, after critical illnesses in older patients [190,191]. It is likely that older patients with severe TBI have enhanced stress responses with high CORT level [179]. In addition, as a response to stress, peripheral aromatization increases with age, resulting in higher E2 levels [192].

After TBI, most women experience disturbed menstrual periods or amenorrhea [193]. If these symptoms, including long-term hormone loss, appear during the reproductive age, it may affect their reproductive ability [194]. In postmenopausal women, increased age at the time of the injury, low hormonal levels, and TBI together can increase the risk of developing dementia [195,196].

It is worth mentioning that trauma induces stress in TBI patients associated with high cortisol (CORT) levels. Importantly, increased CORT levels can lead to HPG axis dysfunction and disturbance in the menstrual cycle and induce hypogonadotropic hypogonadism and amenorrhea in women [197,198]. HPG dysfunction and amenorrhea have also been reported in patients several months after spinal cord injuries [199]. For instance, hypogonadotropic hypogonadism caused by a traumatic injury is a common observation after TBI [200]. In a study by Ranganathan et al., acute and long-term hormonal changes were analyzed in women after severe TBI [182]. They found that increased CORT levels after TBI physiological stress led to anovulation and HPG axis suppression [182]. In another study, similar observations were found in young women: higher injury severity was associated with higher CORT and lower FSH, LH, and E2 levels [201].

These results suggest that high CORT in TBI can induce impairments in HPG axis function, with a consequent reduction of endogenous E2 levels. Low E2 levels may also contribute to neuronal impairments after TBI. However, further experiments and clinical studies are required to elucidate the role and mechanism of TBI in HPG dysfunction.

## 8. Potential Therapeutic Interventions with Estrogenic Compounds in TBI

A long range of estrogen-containing treatment options are available, beginning from oral contraceptives (OCs) to menopausal hormonal replacement therapy (HRT). Menopause is associated with symptoms such as heat waves, mood swings, osteoporosis, the risk of developing heart disease, decreased cognitive function, etc., which have an impact on the quality of life [202,203]. Nowadays, HRT includes several different compounds to reduce these disturbing symptoms; however, studies have shown that the use of E2 alone or in combination with other compounds can lead to unfavorable side effects, such as cardiovascular problems and increased risk of stroke and breast cancer [204,205,206]. Initially, women who received HRT in their early postmenopausal stage have a lower chance of developing cognitive deficits and dementia [207,208,209]. However, several years after HRT, the risk of dementia increases. There is a high possibility of a therapeutic window for the neuroprotective effects of estrogen, which depends on the patient’s age and treatment initiation time after menopause.

Besides the shortcomings of HRT, the use of estrogenic compounds is also arguable in TBI. Most animal studies investigating the neuroprotective effects of estrogen employ E2. Since chronic administration is required for long-term neuroprotection and functional recovery after TBI, the pharmacological development of estrogen-like compounds, which maintain the positive neuroprotective effects of E2 but with none or minimal negative side effects, would be necessary. A recent advance in this line of research was the identification of the promising compound group “Activator of Non-Genomic Estrogen Like Signaling” (ANGELS), such as 4-estren-3α,17β-diol (estren) [210,211]. Estren mimics the neuroprotective effects of E2 in in vivo mouse NMDA-induced toxicity [212] and a β_1–42_-induced neurotoxicity model, while not impacting reproductive tissues [213].

Numerous studies have shown that phytoestrogens such as genistein and formononetin have neuroprotective effects in different animal models of brain injuries both in vitro and in vivo [214,215,216]. The most studied phytoestrogens are genistein and daidzein. Genistein has neuroprotective effects in a rat model of TBI (Marmarou’s) through attenuation of the BBB disruption and prevention against cerebral edema formation [214]. Genistein also reduces kainic acid-induced neuronal cell death in the hippocampus [215]. Formononetin has antitumor, anti-inflammatory, and anti-dyslipidemia effects [217,218]. In a Feeney’s weight-drop model, formononetin promoted neuronal proliferation by the activation of interleukin-1 (IL-10) expression in cortical neurons to inhibit neuroinflammation [219]. These compounds exert their ameliorative actions by protecting the DNA and normal mitochondrial functions [220]. They also exert their anti-inflammatory effects by inducing the expression of different antioxidant enzymes [221,222], providing a promising platform for the treatment of TBI.

Selective estrogen receptor modulators (SERMs) are synthetic molecules with remarkable neuroprotective potential [223]. The first SERMs, such as tamoxifen (TMX) and toremifene, are used in the treatment of breast cancer. TMX can act as an ERα antagonist and agonist [224]. TMX crosses the BBB [225] and exerts an E2-like neuroprotective effect in the brain without the peripheral side effects observed in estrogen therapy [226]. TMX treatment decreases the neuronal apoptosis and cerebral infarction volumes by reducing interleukin-1B (IL-1B) production and increasing the neuronal phosphorylated extracellular signal-regulated kinases 1 and 2 (p-ERK1/2) and B-cell lymphoma 2 (Bcl2) expressions [227]. Studies using a rat LFP model [228] demonstrated that the neuronal ERα-caspase-3 pathway mediates the neuroprotective action of TMX, suggesting a possible role of TMX in therapeutic interventions for human TBI. Bazedoxifen (BZA), a new-generation SERM molecule, was developed for the treatment of osteoporosis in postmenopausal women [229], with favorable effects on the lipid metabolism and skeleton but without uterus or breast stimulation [230]. In vivo experiments demonstrated that BZA suppressed the TBI-induced activation of the mitogen-activated protein kinase/ nuclear factor kappa B (MAPK/NF-κB) signaling pathway [231]. Furthermore, the BZA treatment reduced BBB damage, functional impairments, and the activation of inflammatory cascades in CCI [231], suggesting that BZA could be a possible option for the treatment of TBI.

Tibolone is a synthetic steroid and a member of the selective tissue estrogenic activity regulators (STEARs) employed in HRT [232]. The 3-OH metabolite of tibolon binds to the ER, while the delta-4 isomer binds to the androgen and progesterone receptors [233]. Tibolone metabolites acting on both the ERα and ERβ reduce oxidative stress and preserve the mitochondrial membrane potential [234,235]. In the CNS, astrocytes are believed to mediate the action of tibolone [236]: in an in vivo experiment, tibolone attenuated the reactive response of the microglia and astrocytes in the brain after a TBI in OVX female mice [237].

## 9. Conclusions

Animal and clinical studies indicate that the pathomechanism of TBI is complex. Injury severity, time after injury (hours to days or months/years), and the E2 level significantly influence the possible pharmacological therapeutic interventions after TBI.

The onset of therapeutic interventions for patients with TBI in clinical care units depends on the time required for patient admission and early diagnosis using CT and MRI. However, based on the molecular events of the neuropathological cascade and TBI timeline, early intervention would be more appropriate for the pharmacological treatment of TBI patients. There are also events of secondary injuries occurring in a delayed manner, so the timing of therapy administration should be adapted to the exact target mechanism of the TBI [238].

Although the results of E2 treatment after TBI in animal models are convincing, clinical studies indicate that E2 use is associated with detrimental effects, such as feminization and increased risk of stroke and tumorigenesis, raising concerns about its overall safety. Estrogenic compounds such as SERMS, phytoestrogens, and ANGELS with selective nonclassical estrogen actions are promising options in the therapeutic management of TBI. Neuroprotective estrogenic compounds may offer a better safety profile than the nonselective E2, thereby avoiding E2′s unwanted side effects. Research must continue to find the optimal therapeutic window, dose, and administration route, with special attention addressed to sex differences, age, onset, type, and severity of the TBI. Once these parameters are established, estrogenic compounds could become novel drugs with vast therapeutic potential in TBI.

## Abbreviations

3NPA	3-nitropropionic acid
ANGELS	activator of non-genomic estrogen like signaling
AMPA	α-amino-3-hydroxy5-methyl-4-isoxazole-propionic acid
Bcl2	B-cell lymphoma 2
BZA	bazedoxifen
BBB	blood brain barrier
CCI	controlled cortical impact injury
CORT	cortisol
CPP	cerebral perfusion pressure
CREB	cAMP-responsive element-binding protein
CT	computed tomography
E2	17β-estradiol
eNOS	endothelial nitric oxide synthase
EPM	elevated plus maze
ERα	estrogen receptor alpha
ERβ	estrogen receptor beta
ERE	estrogen response element
ERK1/2	extracellular signal-regulated kinase 1/2
FPI	fluid percussion injury
FSH	follicle stimulating hormone
GFAP	glial fibrillary acidic protein
GPER1	G protein-coupled estrogen receptor 1
GSK3β	glycogen synthase kinase 3 beta
HPG	hypothalamic-pituitary-gonadal
HRT	hormonal replacement therapy
ICP	intracranial pressure
KNDy	kisspeptin, neurokinin B and dynorphin
LPF	lateral fluid percussion injury
LH	luteinizing hormone
MAPK	mitogen-activated protein kinase
MFP	midline fluid percussion injury
MPP	1,3-Bis(4-hydroxyphenyl)-4-methyl-5-(4-(2-piperidinylethoxy)phenol)-1H-pyrazole hydrochloride
MRI	magnetic resonance imaging
MWM	Morris water maze
NF-κB	nuclear factor kappa B
NMDA	N-methyl-D-aspartic acid
OCs	oral contraceptives
OVX	ovariectomized
P2X7	P2X purinoceptor 7
p-ERK1/2	phosphorylated extracellular signal-regulated kinase 1 and 2
PI3K	phosphatidylinositol 3-kinase
PHTPP	4-(2-Phenyl-5, 7-bis (trifluoromethyl) pyrazolo (1,5-a) pyrimidine-3- yl) phenol
SERMs	selective estrogen receptor modulators
ROS	reactive oxygen species
STEARs	selective tissue estrogenic activity regulators
TBI	traumatic brain injury
TNF-α	tumor necrosis factor alpha
TMX	tamoxifen
VGCCs	voltage-gated calcium channels
